# Function of ankle ligaments for subtalar and talocrural joint stability during an inversion movement – an in vitro study

**DOI:** 10.1186/s13047-019-0330-5

**Published:** 2019-03-18

**Authors:** Lu Li, Albert Gollhofer, Heinz Lohrer, Nadja Dorn-Lange, Guiseppe Bonsignore, Dominic Gehring

**Affiliations:** 1 Department of Sport and Sport Science, University of Freiburg, Schwarzwaldstraße 175, 79117 Freiburg, Germany; 2 ESN – European Sportscare Network, Borsigstraße 2, D-65205 Wiesbaden, Germany; 3 Lilium Klinik, Borsigstraße 2, D-65205 Wiesbaden, Germany; 4 Johannes Gutenberg Universität, Mainz, Germany

**Keywords:** Lateral ankle ligament complex, Ankle stability, Ankle inversion restriction, Biomechanics

## Abstract

**Background:**

The lateral ankle ligament complex consisting of the anterior talofibular ligament (ATFL), the calcaneofibular ligament (CFL) and the posterior talofibular ligament (PTFL) is known to provide stability against ankle joint inversion. As injuries of the ankle joint have been reported at a wide range of plantarflexion/dorsiflexion angles, the aim of the present study was to evaluate the stabilizing function of these ligaments depending on the sagittal plane positioning of the ankle joint.

**Methods:**

Eight fresh-frozen specimens were tested on a custom-built ankle deflection tester allowing the application of inversion torques in various plantarflexion/dorsiflexion positions. A motion capture system recorded kinematic data from the talus, calcaneus and fibula with bone-pin markers during inversion movements at 10° of dorsiflexion, at neutral position and at plantarflexion 10°. ATFL, CFL and PTFL were separately but sequentially sectioned in order to assess the contribution of the individual ligament with regard to ankle joint stability.

**Results:**

Joint- and position-specific modulations could be observed when the ligaments were cut. Cutting the ATFL did not lead to any observable alterations in ankle inversion angle at a given torque. But subsequently cutting the CFL increased the inversion angle of the talocrural joint in the 10° plantarflexed position, and significantly increased the inversion angle of the subtalar joint in the 10° dorsiflexed position. Sectioning of the PTFL led to minor increases of inversion angles in both joints.

**Conclusions:**

The CFL is the primary ligamentous stabilizer of the ankle joint against a forced inversion. Its functioning depends greatly on the plantar−/dorsiflexion position of the ankle joint complex, as it provides the stability of the talocrural joint primarily during plantarflexion and the stability of the subtalar joint primarily during dorsiflexion.

**Electronic supplementary material:**

The online version of this article (10.1186/s13047-019-0330-5) contains supplementary material, which is available to authorized users.

## Introduction

Epidemiological data indicate that ankle injuries represent the most frequent type of sport-related injuries [1]. There exists overwhelming evidence that most of the ankle injuries affect the ligaments [2] and that approximately 85% of those injuries are lateral ankle sprains [3]. The aetiology for injuries of the lateral ankle joint typically comprises excessive inversion, often combined with a pronounced plantarflexion and internal rotation of the ankle joint complex [4]. It has been shown that an initial sprain can impair the function of the ankle joint complex in the long term, which is referred to as chronic ankle instability [5].

From an anatomical perspective, the lateral ankle ligament complex consists of the anterior talofibular ligament (ATFL), the calcaneofibular ligament (CFL) and the posterior talofibular ligament (PTFL). Epidemiologic surveys indicate that the ATFL is the ligament that is injured in 85% of all ankle sprain ligament injuries, while the CFL is involved in 35% and the PTFL in 12% [6]. In trying to identify why the individual ligaments are affected during an ankle sprain with different rates, their functional roles and their mechanical properties must be taken into consideration. Most of the current information about the functioning of the ankle ligament originates from in-vitro studies, where custom-made devices were developed to study the biomechanics of the ankle joint while analysing the differential functional importance of the ligaments. Researchers typically applied inversion-eversion stimuli and/or plantarflexion-dorsiflexion in predefined positions of the ankle joint. The biomechanical function is then studied by dissecting a specific ligament and estimating its contribution to stabilizing the ankle joint [7–11].

Increased knowledge about the function of the specific ligaments revealed that the ATFL seems to be the most affected ligament [12, 13], because its functional role is to restrict both plantarflexion and inversion movements [14] and because the maximal tension until failure is quite low [8]. Moreover it has been shown that a certain inversion torque, applied at the fixed foot, increases the posterior tibial displacement by a factor of 2 when the ATFL is dissected [15]. In addition to the ATFL, the CFL seems to resist ankle inversion, as cutting this ligament led to an increased range of inversion [9]. Applying an inversion loading following a dissection of both ATFL and CFL respectively increased the range of motion and decreased the end-range stiffness when compared with the intact and solely ATFL-sectioned ankles [16]. Furthermore, evidence exists showing that a dissection of the CFL causes significant range of motion changes regarding all three planes in the subtalar joint [10]. Thus, it seems that the CFL plays a key role in the lateral stabilization of both the ankle and the subtalar joint. The functional properties of the PTFL were analysed by Ozeki and Kitaoka, who concluded that the PTFL is an important stabilizer especially when the ankle was in a dorsiflexed postion [17]. Previous research has also shown that the strain of the PTFL increases in dorsiflexion (DF) and plantarflexion (PF), but is only minimally affected by ankle inversion [18]. In summary, the PTFL seems to play a supplementary role in lateral ankle stability, especially when the ATFL and CFL are intact [19].

Although it is possible to draw important conclusions about the ligament function from the above mentioned in-vitro studies, specific methodological restrictions have to be considered. First, some of the papers selectively focused on only one specific ligament of the ankle and its defined functional role [9, 14, 15, 18, 19] while others investigated multiple ligaments but considered only coronal movements [7, 16]. Secondly, most of the studies tested the lateral ankle ligament complex with a distinct torque but only either in sagittal or in coronal plane [7, 10, 15, 18, 20, 21]. Yet, investigations of the injury mechanism have revealed that ankle sprains can occur at a wide range of plantarflexion/dorsiflexion angles [4, 22]. It therefore appears important to evaluate the functional properties of the involved ligaments under different plantarflexion/dorsiflexion positions. Third, most of the investigations focused on either the stability of the talocrural joint or on the stability of the subtalar joint alone [7, 15, 17, 18, 21]. Integrative investigations analyzing the function of the ligaments on both joints of the ankle joint complex, and possible interactions, are missing. Finally, in many of the studies, the researchers have tried to slowly rotate the ankle joint to explore the individual ligament’s contribution to ankle joint stability in inversion simulations. For example, investigating the contribution of the CFL, Kobayashi applied an inversion motion that was limited to up to 1°/s [10]; other studies do not even mention the deflection velocity [7, 8, 11]. This may be problematic because it is known that ligament stiffness is related to the speed of its elongation [23] and that typically the ligaments are loaded at high angular velocities during ankle sprains [13].

Therefore, the major purpose of the present study was to evaluate the biomechanics of the talocrural and subtalar joints when the ligaments of the lateral ankle joint complex are sequentially resected. Specifically, we aimed to assess how the sagittal positions would influence the relative contributions of the individual ligaments to joint stability during dynamic inversion.

## Methods

### Specimens

Fourteen fresh-frozen human anatomic lower limb specimens, amputated above the knee joints, were selected for the experiments. Already during the experiments, it became evident that fixation of bone-screws was loosened during dynamic movements in six specimens. Finally, eight cadavers could be included in the study (age: 84.0 ± 5.6 years; donors were two men and two women). Before preparation and measurement, the specimens were kept at room temperature for 24 h in order to ensure conditional physical properties. An experienced anatomist inspected the ankles by manual examination for normal function and confirmed that there was no sign of pathological limitations on any of the cadaver legs. Thereafter, all ankles were exposed layer by layer creating a skin and soft tissue window (5 × 5 cm) distal to the lateral malleolus and ankle. The integrity of the anterior and posterior talofibular ligaments (ATFL and PTFL) and the calcaneofibular ligament (CFL) was verified by visual inspection. During the entire measurements the specimen were regularly irrigated with saline, preventing them from drying-out. The procedures were in accordance with the Declaration of Helsinki and approved by the local ethics committee (#10006/18).

### Experimental equipment setup

**Fig. 1:**
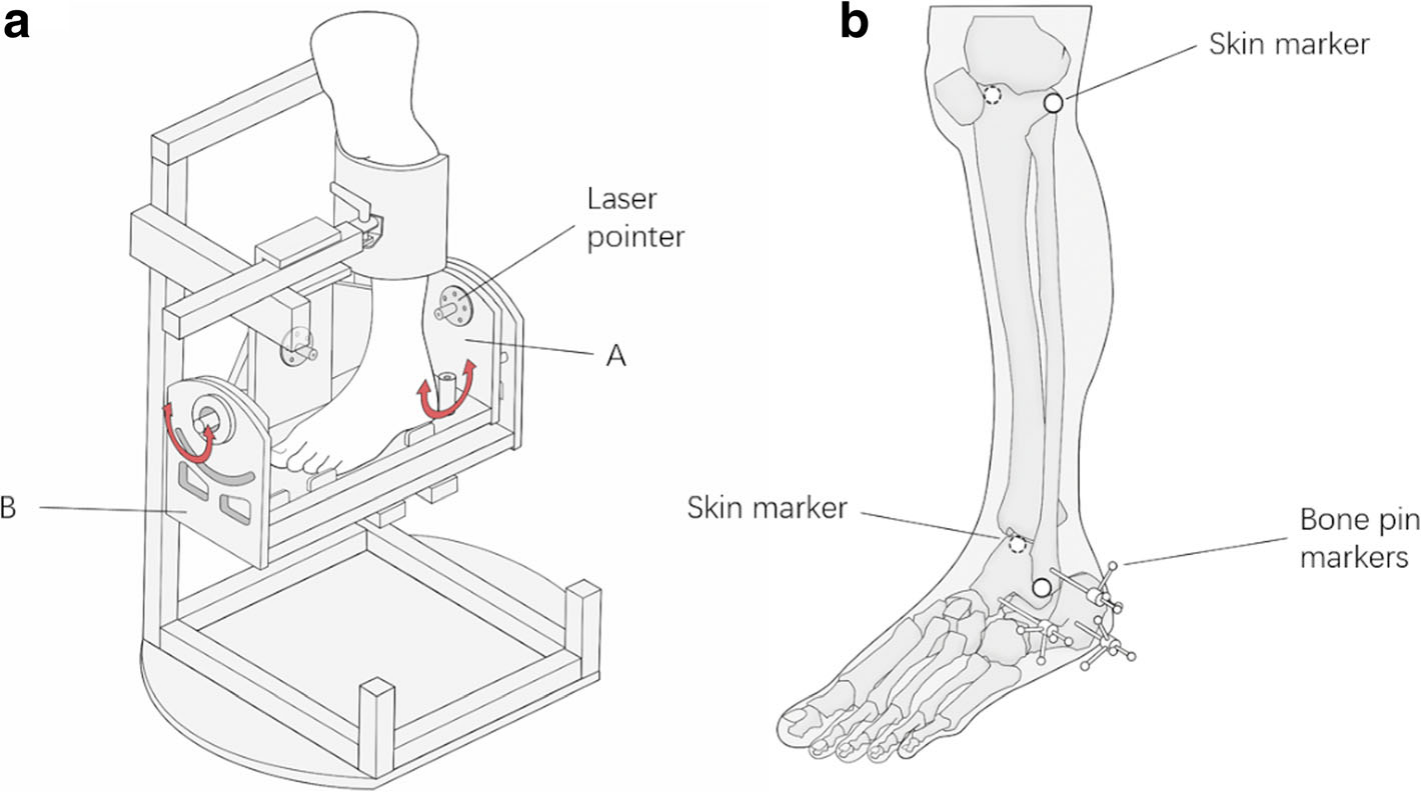
**a** Illustration of the ankle deflection tester (ADT). The apparatus consists of two rotational components on a fixed stand: A) a small fixable tilting platform which can tilt in the coronal plane, B) a bigger flexing platform which can rotate in the sagittal plane. Two laser pointers were installed at the center of rotation of the coronal and sagittal planes to enhance the accurate positioning of the specimen within the ADT. **b** The shin markers were placed at the medial epicondyle of the knee, lateral epicondyle of the knee, medial malleolus and the lateral malleolus. Bone pins with markers were inserted into the fibula, calcaneus and talus

A custom-built ankle deflection tester (ADT) allowed application of inversion torques in various plantarflexion/dorsiflexion positions (Fig. 1a). Mechanically, the ADT consisted of a leg fixation and a foot plate that allowed for independent rotations around the ankle joint in the sagittal (up to ±30°) and frontal planes (up to ±40°). Fixation of the specimen’s foot on the foot plate was secured by applying two non-elastic belts covering the mid-foot and the forefoot (Fig. 1a). Additionally, the leg was fixed to a height-adjustable clamp with straps to ensure stabilization of this segment during inversion movements. To adapt the ADT to the specimen’s individual morphological characteristic, the foot plate was adjusted in height and anterior-posterior position setting, allowing the mechanical rotation axis of the ADT to coincide with the specimen’s talocrural joint. Two goniometer sensors (MODEL 157, Vishay, USA) recorded the angular information of each component along the two mechanical axes of the ADT. A torque meter (TA 125–350, ME-Meßsysteme GmbH, Germany) with a handle was connected with the anterior-posterior axis of the ADT to manually apply and measure the inversion torque.

To record the skeletal motion of the calcaneus, talus and fibula, three Kirschner wires (1.5 mm) were drilled into the lateral aspect of each bone segment, each of them being equipped at its ends with 4 reflective markers (6 mm), building a three-dimensional Cartesian coordinate system (see Fig. 1b). By means of a motion capture system with 11 cameras, the data of each segment in three-dimensional space were tracked at 200 Hz (Vicon Motion System, UK). Additionally, skin markers were placed at the medial and lateral epicondyle of the knee as well as at the medial and lateral malleolus in order to define the anatomical segments in accordance with the recommendation of the ISB (International Society of Biomechanics) [24]. Synchronized with the motion data, the goniometer signals of the ADT were captured with Vicon Nexus 2.5 (Vicon Motion System, UK) at 1000 Hz.

### Experimental procedure

To assess the contribution of each individual ligament on ankle stability an experimental setup was selected in which ligaments were successively cut and the remaining resistance of the ankle joint against external force application was measured. We started with the intact condition. Then, we performed a fixed order of sequentially cutting the ligaments: we first cut the ATFL, then the CFL and finally the PTFL as it is known that the ATFL is typically the first or even only ligament being injured [12], followed by the CFL, and only in rare cases by the PTFL [25]. During each session, specimens were tested in three different positions in the sagittal plane: neutral position, 10° dorsiflexion and 10° plantarflexion. Manually, torque was applied at the ADT in the coronal plane resulting in a sustained inversion-eversion movement. Each simulation trial required 10 inversion-eversion cycles at approximately 1 Hz, in which an inversion angle of 30° had to be reached. The signal of the torque meter and goniometer sensors from the ADT as well as kinematical data of the markers were recorded synchronously.

### Data analysis

The kinematics of the relative bone-to-bone movement between the calcaneus, talus and fibula was derived following the recommendations for ankle joint biomechanics of the International Society of Biomechanics(ISB) [24] using custom written scripts (Bodybuilder, Vicon Motion Systems, UK). Specifically, the four skin markers were used to define the joint centres and orientation of the anatomical coordinate systems, while the bone-pin markers were used to track the skeletal motion. To obtain the angle between fibula and calcaneus, talus and fibula (representing the talocrural joint), and calcaneus and talus (representing the subtalar joint), a Joint Coordinate System approach was used allowing the calculation of the intersegmental movement in 6 degrees of freedom. The inversion/eversion rotation occurs about a floating axis, while the rotation axes for plantar-/dorsiflexion and internal/external rotation occur around body-fixed axes of the proximal or distal segment, respectively. As the ISB recommendation for the ankle joint complex does not differentiate between the talocrural and the subtalar joint, the anatomical coordinate system of the talus was set to correspond to the coordinate system of the calcaneus. Before performing the calculations, all marker trajectories were filtered through a low-pass 2nd order Butterworth filter at 10 Hz.

Aiming to assess the joint stiffness properties in the different measurement conditions, the amount of inversion rotation at a given torque value was extracted. As pre-analysis of the data indicated that irrespective of ligament sectioning the joint stiffness was highly specimen-specific, different torques were needed to induce comparable inversion rotations of the ankle joints between specimens. In order to account for these specimen-specific differences, individualized torque thresholds were defined after pre-analysis using the following approach: For each specimen and throughout all measurement conditions the lowest observable torque value to induce 30° of inversion was extracted. In order to stay always in the linear region of the torque-angle relationship, 90% of this observed torque was set to assess the associated inversion of the ankle joint complex, the talocrural joint and the subtalar joint for all measurement conditions in each specimen. The data extraction was performed with a custom-written script in Matlab (The Mathworks, Natick, MA, USA). For every condition, five continuous inversion-eversion cycles were used for further statistical analysis.

### Statistics

The inversion angles of the ankle joint complex, the talocrural joint and the subtalar joint were considered for statistical analyses. The results of different ligament sectioning conditions were compared using a Friedman test (IBM SPSS statistics 24, USA) in each ankle positions (neutral position, 10° plantarflexion, 10° dorsiflexion). The effect size was calculated using Kendall’s W and values > 0.1 were interpreted as small effects, values > 0.3 as moderate and values > 0.5 as large effects. To show the magnitude of our observation the Wilcoxon test was applied as a post-hoc test to determine the difference between two separate conditions. The results were adjusted by the Bonferroni-Holm correction method. For all tests, *P* < 0.05 was considered as significant.

## Results

### Ankle joint complex

**Fig. 2:**
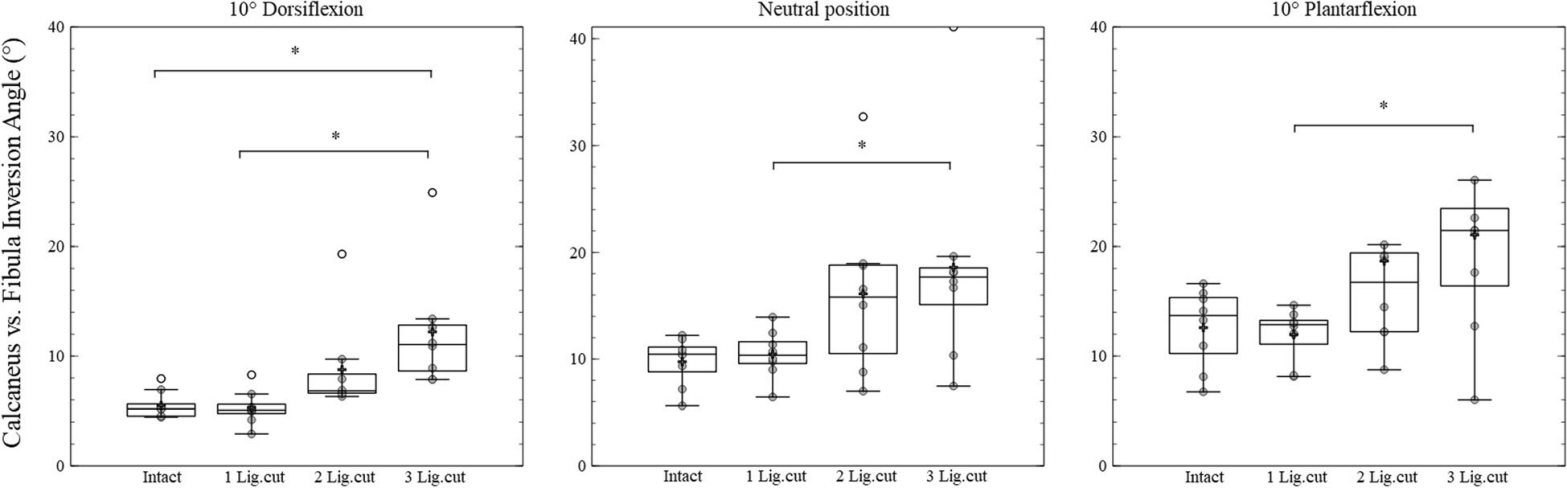
Box-plots including arithmetic mean values (indicated by +) of the ankle joint complex inversion in 10° dorsiflexion, neutral and 10° plantarflexion position. The results of an intact session (Intact), ATFL cut session (1 Lig.cut), ATFL and CFL cut session (2 Lig.cut), ATFL, CFL and PTFL cut session (3 Lig.cut) are shown in each figure. The significant differences between sessions are marked by *

For the inversion angle of the ankle joint complex, main condition effects were observed for all plantarflexion/dorsiflexion positions (at 10° dorsiflexion: *p* = 0.001, effect size = 0.631; in neutral position: *p* = 0.009, effect size = 0.382; in 10° plantarflexion: *p* = 0.013, effect size = 0.362). Post-hoc analysis revealed that at the 10° dorsiflexed angle, cutting all three ligaments significantly increased the inversion angle compared to the conditions when all ligaments were intact (*p* = 0.015) and when the ATFL was cut (*p* = 0.015). In the neutral and in the 10° plantarflexed position a significantly increased inversion was observed when all ligaments were cut compared to the condition when only the ATFL was cut (*p* = 0.015 and *p* = 0.045; Fig. 2).

### Talocrural joint

**Fig. 3:**
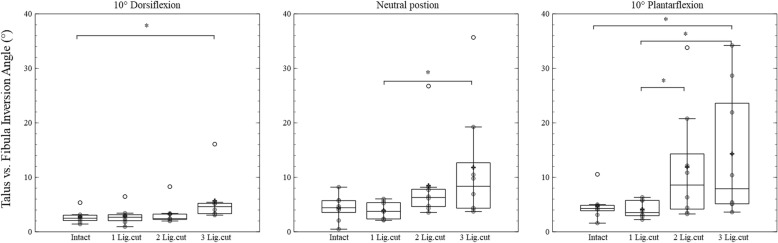
Box-plots including arithmetic means (indicated by +) of the inversion of the talocural joint in 10° dorsiflexion, neutral and 10° plantarflexion position. The results of intact session (Intact), ATFL cut session (1 Lig.cut), ATFL and CFL cut session (2 Lig.cut), ATFL, CFL and PTFL cut session (3 Lig.cut) are shown in each figure. The significant differences between sessions are marked by *

Regarding the talocrural joint, main ligament condition effects were detected for all three positions (at 10° dorsiflexion: *p* = 0.005, effect size = 0.475; in neutral position: *p* = 0.004, effect size = 0.425; at 10° plantarflexion: *p* = 0.001, effect size = 0.598). Positioned at 10° dorsiflexion, cutting all three ligaments led to a significantly increased inversion angle (*p* = 0.015). In the neutral position, dissecting all ligaments was different from the condition when only the ATFL was cut (*p* = 0.015). At 10° of plantarflexion, cutting all the ligaments significantly increased the inversion angle compared to the condition when no ligament was sectioned (*p* = 0.015). Moreover, dissecting the CFL and the PTFL caused a significant increase of the inversion angle compared to the condition with a section of the AFTL alone (*p* = 0.026 and *p* = 0.015, see Fig. 3).

### Subtalar joint

**Fig. 4:**
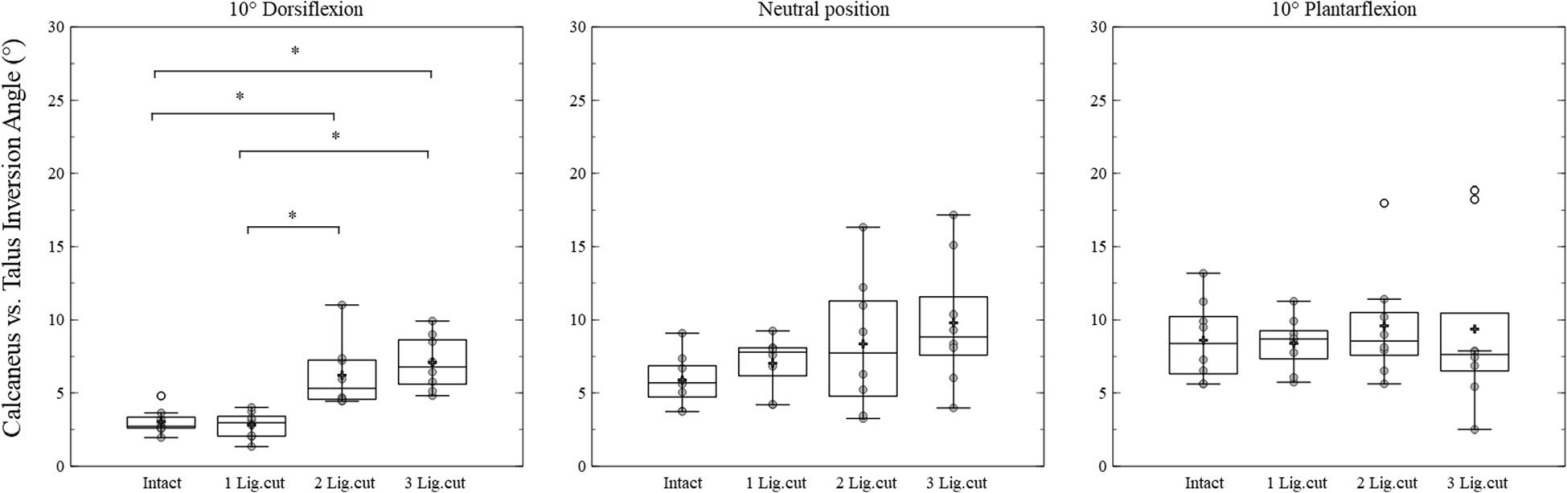
Box-plots including arithmetic mean values (indicated by +) of the inversion of the subtalar joint in 10° dorsiflexion, neutral and 10° plantarflexion position. The result of intact session (Intact), ATFL cut session (1 Lig.cut), ATFL and CFL cut session (2 Lig.cut), ATFL, CFL and PTFL cut session (3 Lig.cut) are shown in each figure. The significant differences between sessions are marked by *

For the subtalar joint, the main ligamentous effects were solely observed in dorsiflexion position (at 10° dorsiflexion: *p* = 0.001, effect size = 0.72; in neutral position: *p* = 0.073, effect size = 0.252; at 10° plantarflexion: *p* = 0.849, effect size = 0.05, see Fig. 4). The post-hoc analysis showed that dissecting ATFL and CFL increased the inversion angle significantly compared to the intact condition (*p* = 0.048). Moreover, compared to the ATFL dissection session, cutting the CFL alone or in combination with the PTFL led to a significant increase of the inversion angle (both *p* = 0.015). Furthermore, cutting all three lateral ankle ligaments leads to a significant increase of the inversion angle (*p* = 0.039). Detailed data of bone-to-bone kinematics can be found in addtional tables in Additional file 1 and Additional file 2.

## Discussion

Using an experimental cadaver approach, our data indicate that the lateral ankle ligaments have different biomechanical functions in stabilizing bony structures during an inversion movement. The motion task of our study (the mean angular velocity is 64.1°/s ± 11.8°/s) was comparable with the previously reported basic movements such as running (maximum ankle inversion velocity: 85.1°/s), cutting (maximum ankle inversion velocity: 37.2°/s) and jumping (maximum ankle inversion velocity: 22.5°/s) [26]. However, our motion task design only reached 25% of an ankle ligamentous sprain simulation, reporting ankle inversion velocities from 200°/s to 300°/s [27]. Depending on the sagittal plane position, the stabilizing function of ligaments changed, especially when not the entire ankle joint complex but rather the sub-structures (talocrural and subtalar joint) were considered. The results support the assumption that when the ATFL is ruptured the main function of the CFL is to stabilize the talocrural joint against inversion in a plantarlexed position, while its primary function during dorsiflexion is most likely to resist the inversion of the subtalar joint. By imitating a lateral ankle sprain and simultaneously investigating the bone-to-bone stabilization directly, this study extends the current knowledge of the function of the lateral ankle ligament complex, providing the following important details:

### Stability of the global ankle joint complex

From a biomechanical perspective, the ankle joint complex consists of the talocrural joint and the subtalar joint and thus can be interpreted as a connection between the tibia/fibula segment and the calcaneus [24]. Regarding the ligamentous structures, the ATFL and CFL should have functional importance in resisting the ankle joint complex both in sagittal and coronal plane [14, 25]. However, the results of the present study reveal that only in situations when all three ligaments were cut, the inversion stability of the entire ankle joint complex is compromised. Our results therefore at least partly confirm earlier experimental data showing that following sectioning the ATFL alone, the maximum inversion motion between calcaneus and tibia in dorsiflexion position was not influenced, whereas sectioning both the ATFL and the CFL significantly increased the inversion motion [28].

Nevertheless, it still remains difficult to understand the function of ligaments in resisting external rotational torques applied to the ankle joint complex if considering the results from the ankle joint complex only. Thus, it seems necessary to separately evaluate the ankle joint complex composing sub-joints, namely the talocrural joint and subtalar joint.

### Stability of the talocrural joint

According to our results, the CFL had a substantial restrictive function for the talocrural joint when the ankle is in 10° plantarflexion position, while at 10°dorsiflexion and in the neutral position, there is no clear evidence for a stabilizing function of the CFL. Specifically, in the plantarflexed position, cutting the CFL additionally to the ATFL increased the inversion angle by 3.3° compared to the situation where solely the ATFL was sectioned. The geometry of the talocrural joint, with its oblique rotation axis and the specific shape of the talus, causes that when the talus is moved in its largest anterior position, the joint was more unstable in plantarflexion [29]. This explains why the stability of the talocrural joint was sensitive to a destruction of the CFL specifically in a plantarflexion positon. Functionally, the CFL constrains the talus through the calcaneus, stabilizing both the talocrural joint and the subtalar joint. The results showed a phenomenon comparable with findings from previous studies observing significantly increased inversion angles after sectioning the CFL significantly during ATFL ruptured conditions [30]. However, based on present data, the stabilizing function of the CFL on the talocrural joint was prominent preferentially in plantarflexion.

In addition, the PTFL could have a potential function in constraining the talocrural joint. When the ATFL and the CFL were already cut, sectioning the PTFL increased the mean inversion angle by 39% (2.1°) in the neutral position and by 75% (2.2°) in dorsiflexion. Even though there was no clear statistical evidence about the contribution of the PTFL, in 10° plantarflexion positions the three ligaments cut condition differed significantly from the other conditions and in 10° dorsiflexion position the same phenomenon was observed. Even in the neutral position, a difference of inversion angle was found between the ATFL cut condition and the three ligaments cut condition. This means that sectioning the complex of lateral ankle ligaments compromises the stabilization of the talocrural joint. In summary, it is concluded that the PTFL contributed to stabilizing the talocrural joint in all three sagittal plane positions. Yet, when interpreting the data carefully, it has to be considered that a specific sequence of sectioning the ligaments was used and the PTFL was always the last ligament that was cut. Thus, our results are comparable to an earlier investigation that the PTFL could not restrict the ankle joint in the inversion movement independently from the other ligaments. This indicates that in an otherwise intact ligamentous complex, the PTFL plays only a supplementary role in ankle stability [19].

### Stability of the subtalar joint

In the subtalar joint, all statistical effects of the ligaments were discovered only in the dorsiflexed position and in general no significant effects were observed in neutral and plantarflexion positions. Referring to the discussion above, one reason for these findings could be that the talocrual joint seems to be stiffened by bony constraints in the dorsiflexed positionand therefore the subtalar joint takes over to lead the motion. In addition, the ATFL was observed loose during a dorsiflexed position in a previous study. This explains why no effects were detected after sectioning the ATFL in dorsiflexion condition [18]. In consequence, the importance of the CFL and the PTFL in contributing to subtalar joint stability is increased in dorsiflexion. Another reason for this observation could be the differences in the orientation of the axes of the subtalar and talocrural joints. Previous studies showed that the inclination angle of the subtalar joint axis was different from that of the ankle joint complex and the talocrural joint [31, 32]. The axes of the sub-joints both differ from the biomechanical axis of the ankle joint complex and this indicates that during an inversion motion the calcaneus might not only rotate in the coronal plane but also internally rotate at dorsiflexion condition.

A previous study reported that specifically a rupture of the CFL would lead to a laxity of the subtalar joint [10]. Furthermore, the CFL was strained and stretched in a dorsiflexion position during an inversion movement [11, 18]. Kobayashi also showed that the CFL tension increased when moving from plantarflexion to dorsiflexion during inversion [30]. Our results support these previous findings. Compared to the ATFL cut condition, sectioning the CFL led to a significant increase of the inversion angle (2.2°) in the dorsiflexion position. This indicated that the dorsiflexion caused a larger stretch between the tibia and calcaneus. Thus, the CFL seems to have a more stabilizing effect when the ankle joint is in dorsiflexion. It is important to remark, that the present study does not allow drawing a direct conclusion about the function of the CFL solely, as a fixed sectioning order was chosen and the ATFL was already sectioned before. Yet, especially in the dorsiflexed position, no changes in ankle joint stability were observed when the ATFL alone was cut. This provides evidence for the important role of the CFL in lateral stabilization of the ankle joint.

### Limitations

Some limitations have to be considered when interpreting the results of the present study. First, the number of specimens might influence the systemic significance of our results. Second, due to the manually induced external torque, minor fluctuations in amplitude and frequency were apparent. In order to minimize this potential error, always the same researcher was requested to rotate the foot plate to the maximum position in every trial. Third, based on the prevalence of ligament rupture, we sectioned the ligaments always in the same sequence and therefore isolated effects of the CFL or the PTFL could not be investigated properly in every positioning condition. A random sectioning sequence could be applied in a bigger sample size in the future to detect the specific stabilizing functions of these structures in isolated conditions. Fourth, the results might be influenced by the age of the specimens and future studies with younger samples are desired.

## Conclusions

The present study suggested that the CFL is the primary ligamentous stabilizer of the ankle joint against a forced inversion. Its functioning depends greatly on the plantar-/dorsiflexion position of the ankle joint complex, as it provides the stability of the talocrural joint primarily during plantarflexion and the stability of the subtalar joint primarily during dorsiflexion.

## Additional files

Additional file 1: Supplementary data 1. (XLSX 9 kb)

Additional file 2: Supplementary data 2. (XLSX 11 kb)

## Abbreviations

ADT: Ankle deflection tester
ATFL: Anterior talofibular ligament
CFL: Calcaneofibular ligament
ISB: International Society of Biomechanics
PTFL: Posterior talofibular ligament
